# The diagnostic value of skin biopsies in Sneddon syndrome

**DOI:** 10.1371/journal.pone.0253365

**Published:** 2021-06-28

**Authors:** N. L. P. Starmans, S. Zoetemeyer, M. R. van Dijk, L. J. Kappelle, C. J. M. Frijns

**Affiliations:** 1 Department of Neurology and Neurosurgery, University Medical Centre Utrecht, Utrecht, The Netherlands; 2 Department of Pathology, Reinier de Graaf Gasthuis, Delft, The Netherlands; 3 Department of Pathology, University Medical Centre Utrecht, Utrecht, The Netherlands; King Faisal University, SAUDI ARABIA

## Abstract

**Background:**

Sneddon syndrome (SS) is defined by widespread livedo reticularis (LR) and stroke. There is no single diagnostic test for SS and diagnosis can be solely based on clinical features. This cross-sectional case-control study aimed to determine the diagnostic value of skin biopsies in SS patients.

**Materials and methods:**

We studied skin biopsies from patients with a clinical diagnosis of SS or isolated LR. We also studied controls with vitiligo or normal skin. Biopsies were considered standardized if 3 biopsies were taken from the white centre of the livedo and reached until the dermis-subcutis border. Biopsies were scored for features of an occlusive microangiopathy without knowledge of the clinical features. Sensitivity and specificity of the biopsy findings were calculated with the clinical criteria as the reference standard.

**Results:**

We included 34 SS patients, 14 isolated LR patients and 41 control patients. Biopsies of 17 patients with SS (50%), 4 with isolated LR (31%) and 10 control patients (24%) showed at least one artery in the deep dermis with a thickened vessel wall combined with recanalization or neovascularization (sensitivity 50% and specificity 69% with LR as reference). Standardized biopsies increased the sensitivity to 70%. In a post hoc analysis the combination of an occlusive microangiopathy and the presence of a livedo pattern in the superficial dermis increased the specificity to 92%.

**Conclusions:**

Standardized skin biopsies can support the clinical diagnosis of SS. An occlusive microangiopathy as the only positive criterion for the diagnosis of SS had insufficient specificity for a definite diagnosis.

## Introduction

Sneddon syndrome (SS) is a rare disease that is defined by the presence of permanent widespread livedo reticularis (LR) and stroke [1–5]. It mostly affects young women [1–5]. Clinical features associated with SS range from neurological symptoms, such as headache or cognitive impairment, to heart valve anomalies and renal insufficiency [2–5].

SS can be a diagnostic challenge, since there is no single diagnostic test and diagnosis is mainly based on clinical features [2]. Brain imaging usually shows cerebral infarcts or haemorrhages in multiple arterial territories and white matter abnormalities [2,4,5]. LR is a prerequisite for the diagnosis of SS [2–5]. However, the extent and severity of livedo is not defined [2–5]. In addition, different types of livedo exist and a physiological subtype of livedo is a common finding, especially in young women [2,3]. Therefore, skin biopsy has been suggested as a helpful diagnostic tool [3,5,6]. It can show abnormalities that may be related to the pathophysiology of SS, such as proliferation of smooth muscle cells of the tunica media and occlusion of the lumen of the small- and medium-sized arteries of the skin [6–8].

Information about skin biopsies in patients with SS is scarce [3–6]. The sensitivity has been described in only one small study and the specificity has not been reported at all [6]. In this cross-sectional case-control study, we investigated skin biopsies of patients with a clinical diagnosis of SS and assessed the diagnostic value of these biopsies.

## Materials and methods

### Study population

**Fig 1:**
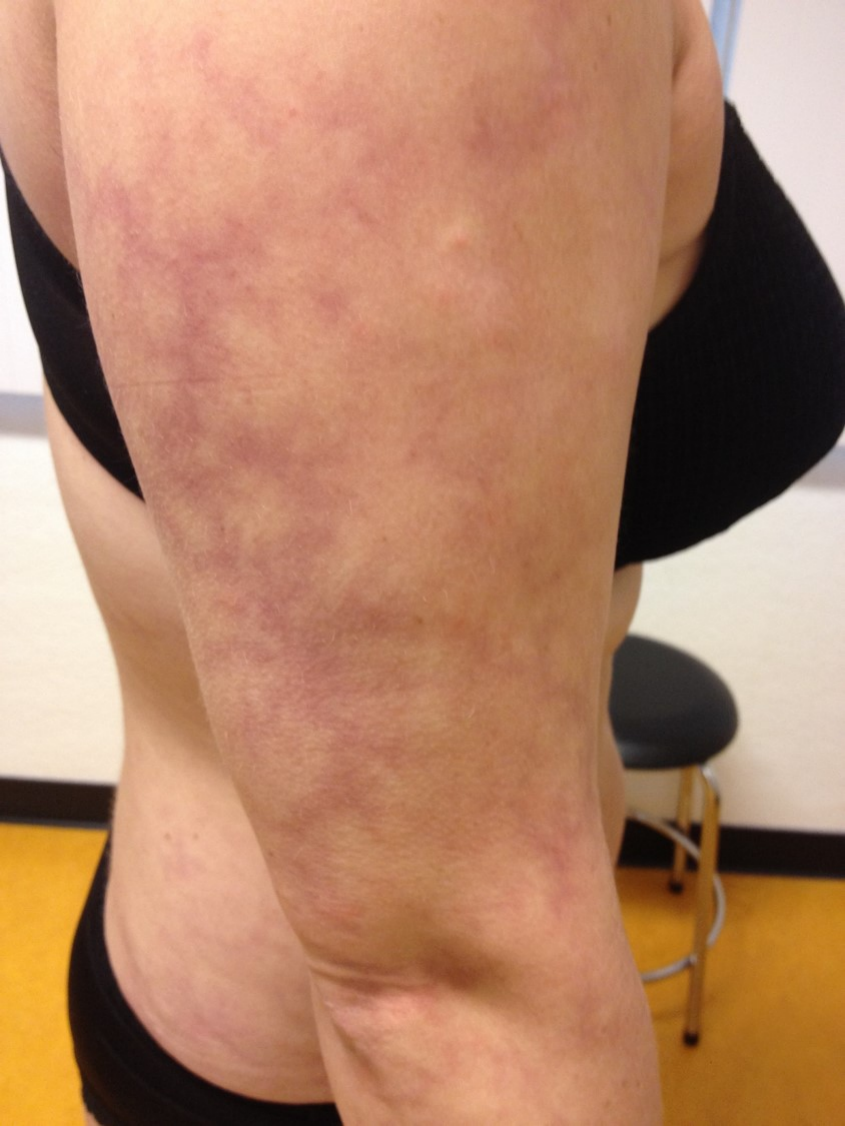
Livedo reticularis. This figure shows an example of a patient with Sneddon syndrome with livedo reticularis on the right arm. This patient also had livedo reticularis on the legs, trunk, buttocks and face.

The Medical Research Ethics Committee of the University Medical Centre Utrecht, The Netherlands, provided a waiver for this study. Informed consent was waived, as this is a retrospective study using anonymized patient data. We did obtain written informed consent from the patient of whom we showed the livedo reticularis in Fig 1.

The biopsy database of the University Medical Centre Utrecht was searched for eligible patients using the following search terms: Sneddon, vasculopathy, livedo, vitiligo and no abnormalities. Patients who underwent skin biopsies between 2000 and 2016 were included if they had a final diagnosis of SS, isolated LR, vitiligo or a histologically normal skin biopsy. LR was defined as a skin abnormality with persistent, erythematous or violaceous, regular unbroken or irregular broken circles [3]. SS was defined as the presence of at least one clinically apparent or silent stroke, visible on brain imaging, with permanent LR involving at least two body locations and other causes of stroke being ruled out after an extensive work-up, including laboratory tests, brain imaging and cardiac evaluation [3,4]. Patients with isolated LR did not have other dermatological abnormalities. The control group consisted of patients with vitiligo, defined as well circumscribed, depigmented macules, and of patients with histologically normal skins [9]. In patients with isolated LR and in controls, history of stroke had to be negative. Patients with systemic lupus erythematosus or vasculitis were excluded from all groups. If the presence of antiphospholipid antibodies (APL) was known, APL-positive patients were excluded from the isolated LR and control group.

### Clinical data

Clinical data were obtained from the patient files by one author (N.S.) at the time of diagnosis in case of patients with SS or at the time of skin biopsy in case of isolated LR or control patients. Baseline characteristics included sex, age at skin biopsy, age at first clinically apparent stroke and age at diagnosis of SS. The presence of LR was recorded in five different locations: face, arms, legs, trunk and buttocks.

The number, subtype and accompanying signs and symptoms of the strokes were recorded. Strokes were classified as ischaemic stroke, haemorrhagic stroke or silent stroke. Silent stroke was defined as an infarct on brain imaging that had not caused neurological deficits. A transient ischaemic attack (TIA) was defined as an acute transient episode of neurological dysfunction caused by focal ischemia of the brain or the retina with clinical features that lasted less than 24 hours [10].

Laboratory parameters included the presence of APL and antinuclear antibodies. Patients were classified as APL-positive if lupus anticoagulant, anticardiolipin antibodies or anti-β2 glycoprotein-1 antibodies were positive at least two times with an interval of at least three months [3,4].

Computed tomography (CT) or magnetic resonance imaging (MRI) at diagnosis was reviewed by one of the authors (N.S.) and in case of doubt by a second author (C.F.). Cerebral infarcts were subdivided into large corticosubcortical (involving > 2 gyri), small corticosubcortical (involving ≤ 2 gyri), subcortical (≥ 15 mm), lacunar (< 15 mm) or cerebellar. Intracranial haemorrhages were subdivided into deep or lobar haemorrhages.

### Histological data

Haematoxylin and eosin stained skin biopsies from the biopsy database were jointly assessed by two dermatopathologists (M.D. and S.Z.), who were blinded for the clinical data. They rated number, depth, location and quality of the biopsies. Quality was considered as good if the biopsy reached at least until the dermis-subcutis border and was not mechanically damaged, as moderate if the biopsy reached at least until the mid-dermis and was not mechanically damaged or as poor if the biopsy reached until the superficial dermis or was mechanically damaged. The presence and severity of SS related abnormalities such as endothelitis, occluded vessels, recanalization, neovascularization, a thickened vessel wall and perivascular inflammation with lymphocytes, were recorded. The presence of a specific livedo pattern, defined as an increased number of vessels and predominantly horizontally arranged vessels in the superficial dermis, was recorded as well.

The biopsies were subsequently marked as definite occlusive microangiopathy, as probable occlusive microangiopathy or as no occlusive microangiopathy. Definite occlusive microangiopathy was defined as a biopsy that showed a thickened vessel wall with partial or total occlusion of the lumen of at least one arteriole or small artery in the deep dermis [6]. Probable occlusive microangiopathy was defined as a biopsy that showed a thickened vessel wall with recanalization or neovascularization of at least one arteriole or small artery in the deep dermis [6]. All other abnormalities were classified as no occlusive microangiopathy.

### Data analysis

Analyses were performed with SPSS Statistics version 25.0. Clinical and histological data are presented as frequencies with percentages of total or median with range. Because of the small sample size, histological data of the different groups were compared with nonparametric tests. Fisher’s exact tests were used to compare dichotomous variables and Mann-Whitney tests were used to compare continuous variables. A p-value of < 0.05 was considered statistically significant. The sensitivity, specificity and corresponding 95% confidence intervals of the skin biopsies were calculated with the previously stated clinical criteria as the reference. Patients with SS served as cases and were compared with both control patients and patients with isolated LR.

We performed additional, not prespecified analyses to determine the optimal skin biopsy technique and diagnostic criteria. For these post hoc analyses, biopsies were considered standardized in patients with SS if three biopsies were taken from the white centre of the livedo and reached at least until the dermis-subcutis border [6]. We also tested a livedo pattern in the superficial dermis and a thickened vessel wall with recanalization or neovascularization in at least one arteriole or small artery in the deep dermis, as positive criteria for SS.

## Results

### Clinical characteristics

We included 34 patients in the SS group, 14 patients in the isolated LR group and 41 patients in the control group. Eighty-eight percent of the patients in the SS group were female with a median age at diagnosis of 40.5 years. The median age at skin biopsy ranged from 42.0 to 52.0 years in all groups (Table 1). LR was most commonly present on the arms, legs and trunk (Fig 1). The median number of locations with livedo was 3 in the SS group and 2 in the isolated LR group.

**Table 1:** Clinical characteristics at diagnosis.

Characteristic	SS (N = 34)	Isolated LR (N = 14)	Controls (N = 41)
	N (%) Median (range)	N (%) Median (range)	N (%) Median (range)
**Baseline data**			
Female	30 (88)	11 (79)	15 (37)
Age at skin biopsy (years)	42.0 (21–66)	52.0 (14–75)	47.0 (21–76)
Age at first vascular event (years)	39.0 (18–62)	-	-
Age at diagnosis (years)	40.5 (21–63)	-	-
**Dermatological data**			
Location of LR			
Total number of locations (n)	3.0 (2–5)	2.0 (1–4)	-
Face	6 (18)	0 (0)	-
Arms	32 (94)	8 (57)	-
Legs	34 (100)	13 (93)	-
Trunk	25 (74)	6 (43)	-
Buttocks	3 (9)	3 (21)	-
**Neurological data**			
Type of index event			
Ischaemic stroke	24 (71)	-	-
TIA	4 (12)	-	-
Amaurosis fugax	0 (0)	-	-
Haemorrhagic stroke	0 (0)	-	-
Cognitive impairment	3 (9)	-	-
Silent stroke	3 (9)	-	-
**Laboratory data**			
APL-positive	11/34 (32)	0/7 (0)	0/3 (0)
ANA	8/25 (32)	3/10 (30)	3/9 (33)
**Radiological data**			
Total number of lesions (n)	4.0 (1–8)	-	-
Cerebral infarcts (n)	120 (100)	-	-
Large corticosubcortical	44 (37)	-	-
Small corticosubcortical	36 (30)	-	-
Subcortical	5 (4)	-	-
Lacunar	10 (8)	-	-
Cerebellar	25 (21)	-	-

ANA, antinuclear antibodies; APL, antiphospholipid antibodies; LR, livedo reticularis; SS, Sneddon syndrome; TIA, transient ischaemic attack.

At the time of diagnosis, the 34 patients in the SS group had had 58 vascular events (38 ischaemic strokes, 19 TIAs and 1 amaurosis fugax). This resulted in a median number of vascular events at diagnosis of 1 (range 0–5). Most patients presented with an ischaemic stroke with symptoms corresponding to the middle cerebral artery territory, such as a sensorimotor deficit, aphasia or central facial palsy (Table 1).

In 30 patients of the SS group an MRI at diagnosis was available, 2 patients underwent a CT and in 2 patients, scans were unavailable for full review. Brain imaging showed cerebral infarcts in all patients with a majority of corticosubcortical infarcts. Multiple arterial territories were involved in 26 (81%) (Table 1). We did not find any haemorrhagic strokes.

### Histology

The median number of punches per skin biopsy was 4.0 in the SS group, 1.5 in the isolated LR group and 1.0 in the control group. The quality of the biopsies was good, except for 1 biopsy in the SS group and 3 biopsies in the isolated LR group, which were of moderate quality. Only 1 biopsy in the isolated LR group was of poor quality and was excluded from the analyses.

**Fig 2:**
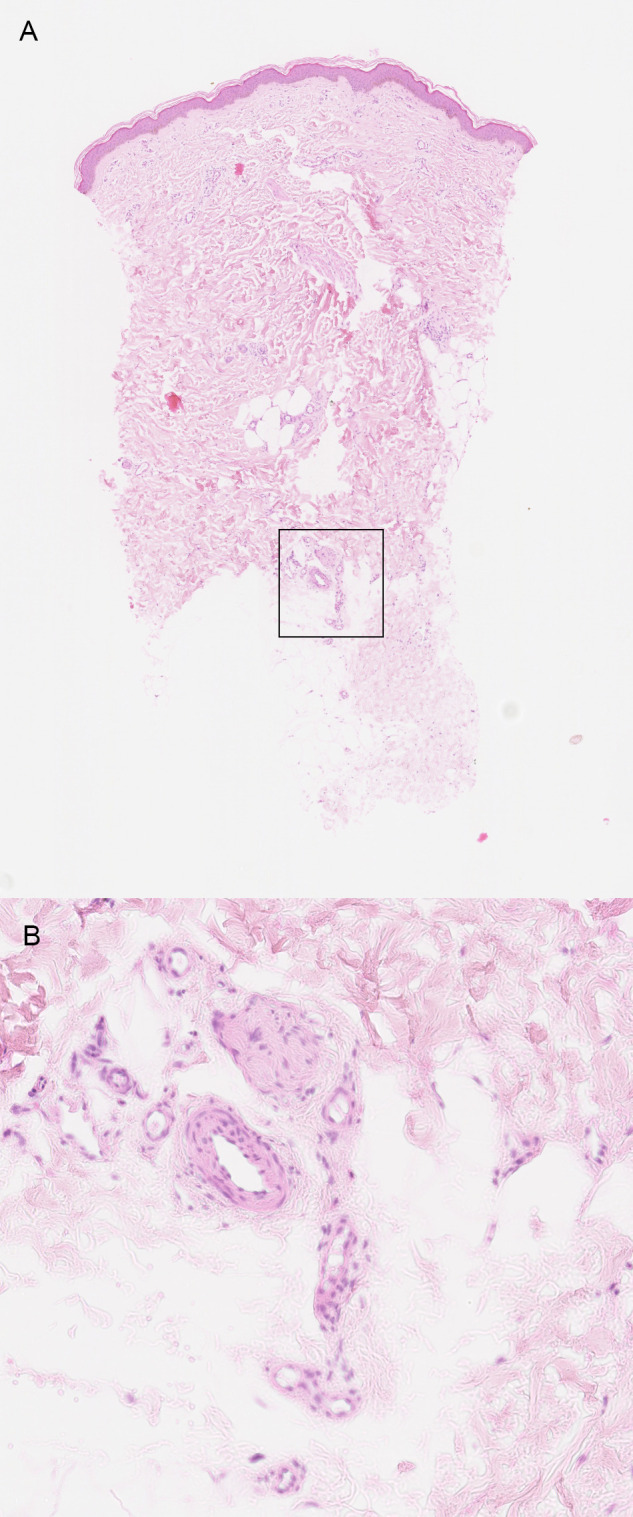
Example one of a probable occlusive microangiopathy. (A) Low magnification shows that the abnormal arteries are located in the deep dermis (box) (H&E, 25x). (B) In detail, multiple arteries with a patent lumen and a thickened vessel wall, suggesting neovascularization (H&E, 100x).

**Fig 3:**
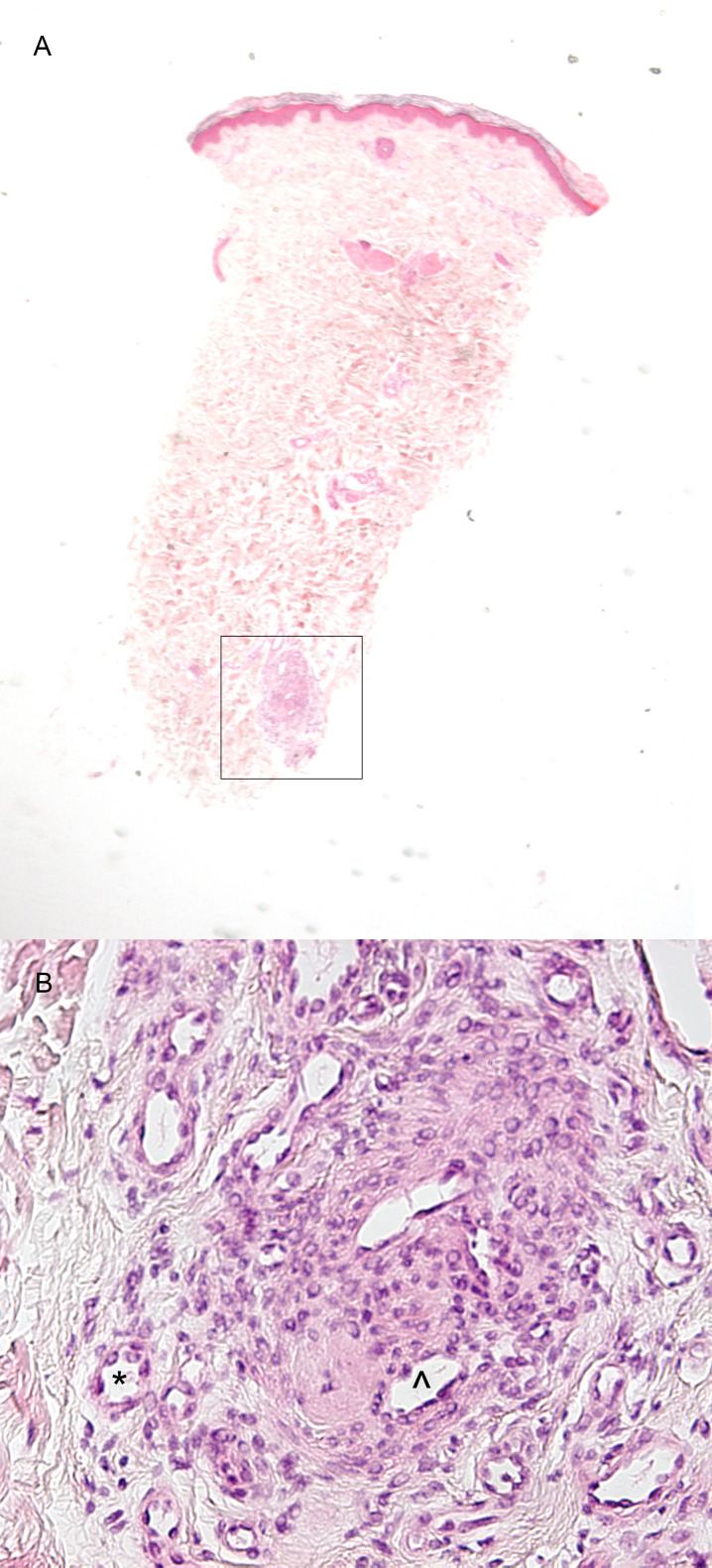
Example two of a probable occlusive microangiopathy. (A) Low magnification shows that the abnormal arteries are located in the deep dermis (box) (H&E, 20x). (B) In detail, extensive neovascularization (*) and recanalization (^) are visible (H&E, 200x).

**Fig 4:**
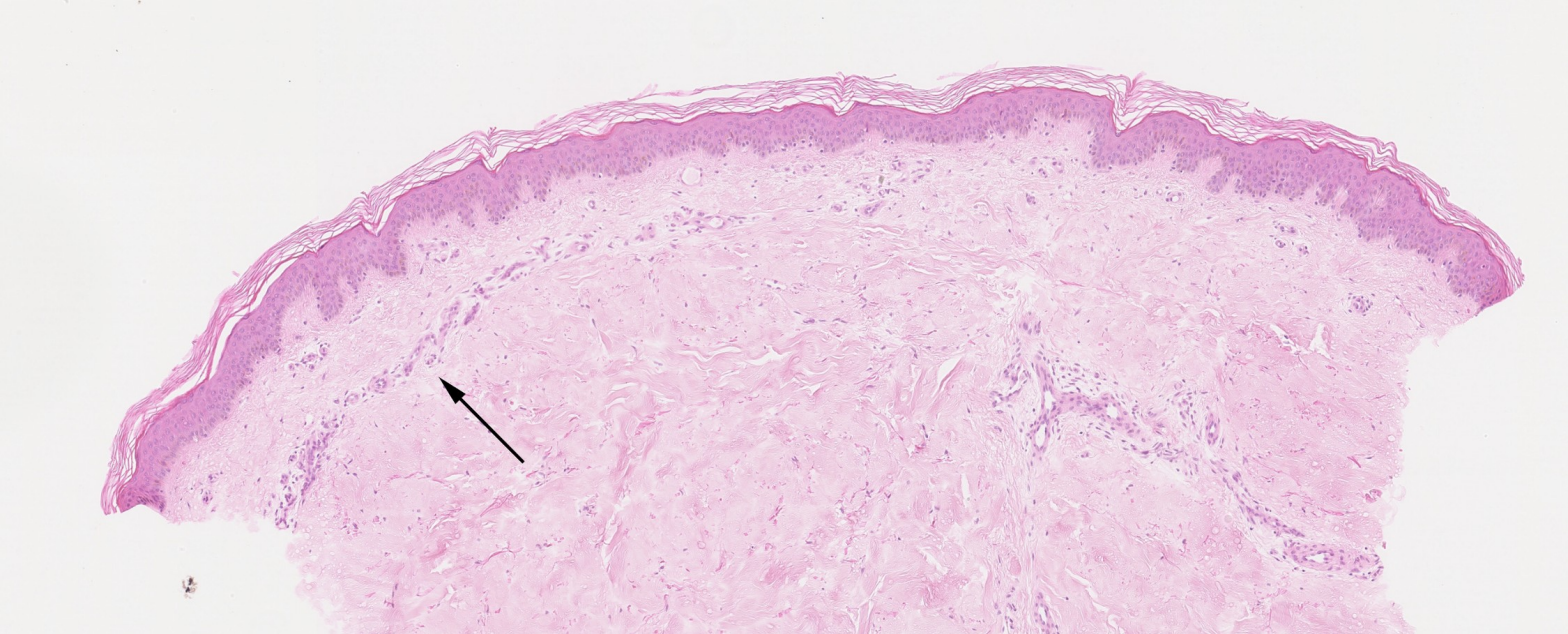
Example of a livedo pattern. The right side of the biopsy shows a normal superficial dermis. On the left side of the biopsy, a livedo pattern is visible (arrow). There are more arteries in the superficial dermis, which are more horizontally arranged (H&E, 50x).

None of the biopsies met the criteria for a definite occlusive microangiopathy. Seventeen SS patients (50%), 4 isolated LR patients (31%) and 10 control patients (24%) had biopsies that fulfilled the criteria of a probable occlusive microangiopathy (Figs 2 and 3). No abnormalities were found in 2 patients in the SS group, in 5 from the isolated LR group and in 8 from the control group. Only 2 patients in the SS group had partially or totally occluded vessels in the deep dermis, the obstruction being cell-rich in one and cell-poor in the other, both without neovascularization or a thickened vessel wall. Other abnormalities in the deep dermis, such as neovascularization or a thickened vessel wall, were more frequently present in the SS group than in the isolated LR or control group. A livedo pattern in the biopsy was found significantly more often in the SS group (59%) than in the isolated LR group (15%) and the control group (15%) (Table 2 and Fig 4).

**Table 2:** Histology findings.

Characteristic	SS (N = 34)	Isolated LR (N = 13)	p-value	Controls (N = 41)	p-value
	N (%)	N (%)		N (%)	
**Standardized biopsy**	10 (29)	-		-	
**Outcome**					
Definite occlusive microangiopathy	0 (0)	0 (0)	1.00	0 (0)	1.00
Probable occlusive microangiopathy	17 (50)	4 (31)	0.33	10 (24)	0.03
**Abnormalities**					
Superficial dermis					
Livedo pattern	20 (59)	2 (15)	0.01	6 (15)	< 0.01
Neovascularization	10 (29)	5 (39)	0.73	16 (39)	0.47
Mild	10 (100)	5 (100)		14 (88)	
Moderate or severe	0 (0)	0 (0)		2 (13)	
Thickened vessel wall	3 (9)	0 (0)	0.55	0 (0)	0.09
Mild	2 (67)	0 (0)		0 (0)	
Moderate or severe	1 (33)	0 (0)		0 (0)	
Perivascular lymphocytes	15 (44)	3 (23)	0.32	16 (39)	0.81
Mild	13 (87)	2 (67)		11 (69)	
Moderate or severe	2 (13)	1 (33)		5 (31)	
Deep dermis					
Endothelitis	0 (0)	0 (0)	1.00	0 (0)	1.00
Occluded vessels	2 (6)	0 (0)	1.00	0 (0)	0.20
Recanalization	2 (6)	0 (0)	1.00	0 (0)	0.20
Neovascularization	20 (59)	5 (39)	0.33	13 (32)	0.02
Mild	11 (55)	2 (40)		9 (69)	
Moderate or severe	9 (45)	3 (60)		4 (31)	
Recanalization or neovascularization	20 (59)	5 (39)	0.33	13 (32)	0.02
Thickened vessel wall	23 (68)	5 (39)	0.10	14 (34)	0.01
Mild	14 (61)	5 (100)		11 (79)	
Moderate or severe	9 (39)	0 (0)		3 (21)	
Perivascular lymphocytes	5 (15)	2 (15)	1.00	3 (7)	0.46
Mild	4 (80)	1 (50)		3 (100)	
Moderate or severe	1 (20)	1 (50)		0 (0)	

LR, livedo reticularis; SS, Sneddon syndrome.

### Diagnostic accuracy–prespecified analyses

Since none of the biopsies showed a definite occlusive microangiopathy, this criterion could not be used. If the definition of a probable occlusive microangiopathy was used as the criterion for positivity, the sensitivity and specificity of the skin biopsies were 50% and 76%, respectively in comparison with the control patients and 50% and 69%, respectively in comparison with the isolated LR patients (Table 3).

**Table 3:** Test characteristics of the skin biopsy.

	SS versus isolated LR		SS versus controls	
	Sensitivity	Specificity	Sensitivity	Specificity
	95% (CI)	% (95% CI)	95% (CI)	% (95% CI)
**Definite occlusive microangiopathy as diagnostic criterion**				
All biopsies	0 (0–10)	100 (75–100)	0 (0–10)	100 (91–100)
Standardized biopsies only	0 (0–31)	100 (75–100)	0 (0–31)	100 (91–100)
**Probable occlusive microangiopathy as diagnostic criterion**				
Diagnostic criteria 1				
All biopsies	50 (32–68)	69 (39–91)	50 (32–68)	76 (60–88)
Standardized biopsies only	70 (35–93)	69 (39–91)	70 (35–93)	76 (60–88)
Diagnostic criteria 2				
All biopsies	29 (15–47)	92 (64–100)	29 (15–47)	100 (91–100)
Standardized biopsies only	70 (35–93)	92 (64–100)	70 (35–93)	100 (91–100)

CI, confidence interval; LR, livedo reticularis; SS, Sneddon syndrome.

Definite occlusive microangiopathy according to both diagnostic criteria 1 and 2: thickened vessel wall with partial or total occlusion of the lumen in at least one arteriole or small artery in the deep dermis.

Probable occlusive microangiopathy according to diagnostic criteria 1 (prespecified): thickened vessel wall with recanalization or neovascularization in at least one arteriole or small artery in the deep dermis. Probable occlusive microangiopathy according to diagnostic criteria 2 (not prespecified): livedo pattern in the superficial dermis and a thickened vessel wall with recanalization or neovascularization in at least one arteriole or small artery in the deep dermis.

### Diagnostic accuracy–additional analyses

Ten biopsies (29%) were standardized in the SS group. Standardization resulted in an increase in sensitivity from 50% to 70%, whereas the specificity did not change (Table 3).

The adjusted set of pathological diagnostic criteria including both a livedo pattern in the superficial dermis and a thickened vessel wall with recanalization or neovascularization in at least one arteriole or small artery in the deep dermis, had a sensitivity of 29% (10/34) and a specificity 100% (41/41), as compared with the control patients. With isolated LR patients as the reference, the sensitivity and specificity were 29% (10/34) and 92% (12/13), respectively. If we analysed only standardized biopsies, the sensitivity of the adjusted set of pathological diagnostic criteria increased to 70% (7/10) in both comparisons without reduction in specificity (Table 3).

## Discussion

This study focused on the histopathology and diagnostic value of skin biopsies in patients with SS. Our main finding was that standardized skin biopsies that showed the combination of at least one artery in the deep dermis with a thickened vessel wall and recanalization or neovascularization can be helpful to differentiate SS from isolated LR (sensitivity 70%, specificity 69%). Occluded vessels were found in a much lower frequency and can therefore not be used as the main diagnostic criterion for SS. The presence of a livedo pattern in the superficial dermis could be a promising new clue for the diagnosis of SS since it increases the specificity to 92%.

The clinical characteristics of the patients in the SS group are in accordance with the previously reported features of these patients [1,3,4,11–16]. At a relatively young age, the patients suffered from one or multiple ischaemic strokes or TIAs, usually in corticosubcortical areas [3,4]. Intracranial haemorrhages are rare [3,4]. The two largest studies including 53 and 46 patients with SS, respectively, considered LR as widespread if it involved the trunk, buttocks, or both [3,4]. However, this definition is quite restrictive as the LR can also be widespread without involving these two locations [1,13,15].

Studies about histological skin findings in SS patients in detail are scarce and the described abnormalities differ between studies. Most authors have described occluded vessels with a thickened wall, caused by the proliferation and subendothelial migration of smooth muscle cells of the tunica media [3,4,7,8,14,15]. The affected vessels are predominantly small- to medium-sized arteries at the border of the dermis and subcutis [3,7,8,14,15]. Some authors also described a perivascular lymphohistiocytic infiltrate [7,8,13–15]. Biopsies did not show any signs of vasculitis [1,13]. We observed similar findings, and additionally showed vascular abnormalities in the superficial dermis.

There are several possible explanations for the variation in the reported abnormalities in skin biopsies. The most probable explanation is patient selection from different departments or based on different clinical criteria across different studies. Another factor may be reporting bias. While some authors report all abnormalities in the skin biopsies, others only report biopsy findings that are considered as ‘definitely SS’. This could lead to an incomplete overview of all abnormalities related to SS. Two studies, including 15 and 18 patients, respectively, proposed that the abnormalities in the skin biopsies should follow a distinct time course, in which all these different biopsy results fit [7,8]. We could not identify all these stages, particularly the first stage of endothelitis, and neither did another study with a large number of skin biopsies [3]. Additionally, endothelitis was found in only a minority of the patients in the studies that initially proposed this hypothesis [7,8]. Therefore, the contribution of endothelitis in the pathophysiology of SS in unclear.

Occluded vessels, neovascularization and a thickened vessel wall are not specific for SS. Although the biopsy results of the SS patients differed from those of the patients with LR only, a substantial number of control patients, without LR or stroke, also had biopsies indicating a probable occlusive microangiopathy. The histological abnormalities might represent a more general process, which may occur in diseases with microvascular damage [3]. This means that skin biopsies are only useful if clinical features fit with a possible diagnosis of SS.

The applied biopsy technique has a substantial impact on the sensitivity and specificity of the skin biopsies. The rationale for the preferred biopsy technique can be found in the vascular anatomy of the skin. Each skin segment receives blood from a centrally located artery of the lower dermal vascular plexus [6,7,17]. A stenosis or occlusion of this artery will lead to hypoxia, which is most severe in the periphery of the skin segment [6,7,17]. This results in the visible violaceous netlike pattern of the skin [6,7,17]. So in order to find the abnormal and causal vessel, one has to perform a deep biopsy of the white centre of the LR instead of the violaceous network [6,7]. The importance of standardized biopsies was first described by a small study including 15 SS patients [6]. In this study, a thickened vessel wall with occluded vessels served as the pathological criterion [6]. Sensitivity increased from 27% with one biopsy to 53% with two biopsies and to 80% with three biopsies, if these were all taken from the white centre of the LR [6]. Similarly, we found that the sensitivity increased to 70% in the analyses including standardized biopsies only.

The optimal criteria for a skin biopsy confirming the diagnosis of SS cannot be established yet. None of the biopsies in our SS patients met the criterion of an occlusive microangiopathy. We feel that this requirement is too strict and that presence of recanalization or neovascularization suggesting a previous occlusion, could be a more useful definition of a positive biopsy.

Our study has several strengths. It is the first study that reports both the sensitivity and the specificity of skin biopsies in diagnosing SS. These biopsies were scored by two dedicated dermatopathologists, who were blinded to the clinical data of the patients, eliminating any potential observer bias. On the other hand, there are also some limitations. First, a reference standard for the diagnosis of SS is lacking. This means another cause of stroke, such as atherosclerosis or a cardiac source of embolism, cannot be completely excluded. Second, the retrospective collection of data had several consequences, such as missing data and a large number of unstandardized biopsies. Third, because of the design of our study, we cannot calculate a meaningful positive and negative predictive value.

## Conclusions

In conclusion, abnormalities in skin biopsies are not specific for SS and should always be interpreted within the appropriate clinical context. Nevertheless, skin biopsies can be helpful in diagnosing SS. We recommend performing standardized skin biopsies in all patients with a differential diagnosis of SS. We suggest that biopsies are regarded as positive if they show at least one artery in the deep dermis with a thickened vessel wall combined with recanalization or neovascularization. Furthermore, the presence of a livedo pattern in the superficial dermis is a promising new clue with the potential of substantially increasing the specificity. Future research should focus on determining the diagnostic value of skin biopsies in a prospective study to confirm our findings.
