# Correction: The diagnostic value of skin biopsies in Sneddon syndrome

**DOI:** 10.1371/journal.pone.0314784

**Published:** 2024-11-27

**Authors:** N. L. P. Starmans, S. Zoetemeyer, M. R. van Dijk, L. J. Kappelle, C. J. M. Frijns

The footnotes are incorrectly formatted in Table 3. The definitions of the two sets of diagnostic criteria should have not been captured as text under the subheading ‘Diagnostic accuracy–prespecified analyses’. Please see the complete, correct Table 3 footnotes here.

**Table 3:** Test characteristics of the skin biopsy.

	SS versus isolated LR		SS versus controls	
	Sensitivity	Specificity	Sensitivity	Specificity
	95% (CI)	% (95% CI)	95% (CI)	% (95% CI)
**Definite occlusive microangiopathy as diagnostic criterion**				
All biopsies	0 (0–10)	100 (75–100)	0 (0–10)	100 (91–100)
Standardized biopsies only	0 (0–31)	100 (75–100)	0 (0–31)	100 (91–100)
**Probable occlusive microangiopathy as diagnostic criterion**				
Diagnostic criteria 1				
All biopsies	50 (32–68)	69 (39–91)	50 (32–68)	76 (60–88)
Standardized biopsies only	70 (35–93)	69 (39–91)	70 (35–93)	76 (60–88)
Diagnostic criteria 2				
All biopsies	29 (15–47)	92 (64–100)	29 (15–47)	100 (91–100)
Standardized biopsies only	70 (35–93)	92 (64–100)	70 (35–93)	100 (91–100)

CI, confidence interval; LR, livedo reticularis; SS, Sneddon syndrome.

Definite occlusive microangiopathy according to both diagnostic criteria 1 and 2: thickened vessel wall with partial or total occlusion of the lumen in at least one arteriole or small artery in the deep dermis.

Probable occlusive microangiopathy according to diagnostic criteria 1 (prespecified): thickened vessel wall with recanalization or neovascularization in at least one arteriole or small artery in the deep dermis. Probable occlusive microangiopathy according to diagnostic criteria 2 (not prespecified): livedo pattern in the superficial dermis and a thickened vessel wall with recanalization or neovascularization in at least one arteriole or small artery in the deep dermis.
